# Screening of microRNAs controlling body fat in *Drosophila melanogaster* and identification of miR-969 and its target, Gr47b

**DOI:** 10.1371/journal.pone.0219707

**Published:** 2019-07-18

**Authors:** William Redmond, Dylan Allen, M. Christian Elledge, Russell Arellanes, Lucille Redmond, Jared Yeahquo, Shuyin Zhang, Morgan Youngblood, Austin Reiner, Jin Seo

**Affiliations:** Department of Biology, School of Arts and Sciences, Rogers State University, Claremore, Oklahoma, United States of America; University of Mississippi, UNITED STATES

## Abstract

MicroRNAs (miRNAs) are small non-protein coding RNAs and post-transcriptionally regulate cellular gene expression. In animal development, miRNAs play essential roles such as stem cell maintenance, organogenesis, and apoptosis. Using gain-of-function (GOF) screening with 160 miRNA lines in *Drosophila melanogaster*, we identified a set of miRNAs which regulates body fat contents and named them microCATs (microRNAs Controlling Adipose Tissue). Further examination of egg-to-adult developmental kinetics of selected miRNA lines showed a negative correlation between fat content and developmental time. Comparison of microCATs with loss-of-function miRNA screening data uncovered miR-969 as an essential regulator of adiposity. Subsequently, we demonstrated adipose tissue-specific knock-down of gustatory receptor 47b (Gr47b), a miR-969 target, greatly reduced the amount of body fat, recapitulating the miR-969 GOF phenotype.

## Introduction

Obesity is one of the most prevalent public health problems and is rapidly escalating worldwide[1,2]. In addition to social and psychological consequences, obesity is a significant risk factor for cardiovascular disease, type 2 diabetes, fatty liver, and cancer[3].

In the early 60s, James Neel introduced the ‘thrifty gene hypothesis’, which provides an evolution-based elegant explanation for the modern obesity epidemic[4,5]. Thrifty genes function efficiently to store energy (fat) to prepare for a famine. However, modern industrialized societies have frequent feasts and rare famines. Thus, selecting for thrift genes becomes maladaptive. Based on a similar hypothesis, obese fruit flies were isolated from a natural population in Kaduna, Nigeria, and the responsible gene, adipose (adp), was later identified and cloned[6,7]. Further, it was shown that adp is conserved from flies to mice to humans[8]. We hypothesized that “obesity genes” exist and exacerbate this obesity epidemic synergistically with behavior factors (too much high-calorie food consumption and little exercise).

MicroRNAs (miRNAs) were first identified in *Caenorhabditis*. *elegans* and later in all metazoans. They are significantly conserved among numerous species including flies, mice, and humans[9–11]. MiRNAs are a family of 21–25 nucleotide small RNAs. They are first transcribed as longer primary miRNA (pri-miRNA) from the cellular genome and then cleaved by miRNA processing proteins (e.g. Drosha, DGCR8) in the nucleus. The cleaved miRNAs are exported to the cytoplasm and ultimately processed to mature miRNA by Dicer. The mature miRNAs are incorporated into a ribonucleotide-induced silencing complex (RISC). Argonaute proteins guided by miRNA in the RISC complex, identify target mRNAs, and induce translational repression and destabilization of the target transcripts[12–14]. MiRNAs have been shown to regulate expression of genes involved in development, cell proliferation and differentiation[15,16]; further, dysregulation of miRNAs can cause multiple diseases including metabolic disorders, cardiovascular diseases, and cancers[17–20].

MiRNAs fine-tune the expression of genes in lipid metabolism and adipogenesis to maintain energy homeostasis[17,21]. Altered miRNA expression can lead to hyperlipidemia, cardiovascular disease, and metabolic disorders[17,18,21]. However, some miRNAs can reduce triglyceride content and inhibit adipogenesis in tissue culture cells[22]. Further, anti-microRNAs have been successfully used to improve insulin sensitivity in diet-induced obese mice[23–25]. However, a complete understanding of mechanisms of miRNAs on adipogenesis and lipid metabolism is far from thorough.

We screened miRNAs which alter the amount of body fat in *Drosophila melanogaster* for the following reasons: (1) the fruit fly genome contains tractable number of miRNAs (258 miRNAs: miRBase V. 22.1); (2) both gain-of-function (GOF) and loss-of-function (LOF) miRNA libraries are available for genetic screening; and (3) the miRNA gene class has been shown to regulate multiple developmental processes. Here, we identified and demonstrated that miR-969, and its target, gustatory receptor 47b (Gr47b) were essential regulators to control body fat in fruit flies. We further established a negative correlation between the amount of body fat and egg-to-adult developmental time.

## Materials and methods

### Fly stocks

All fly stocks and mates were maintained in Nutri-Fly BF food (Genesee Scientific) with 12 hour day/ 12 hour night cycles at 23°C. UAS-microRNA, UAS-miR-969 sponge, microRNA knock-out, Lsp2-Gal4, UAS-Gr47b RNAi, UAS-Gr10b RNAi, UAS-Gr59e RNAi, UAS-Gr59f RNAi, and W^1118^ lines were purchased from Bloomington *Drosophila* Stock Center. Act5C-Gal4, nSyb-Gal4, and Dcg-Gal4 drivers were gifts from Dr. John P Masly (University of Oklahoma) and Dr. Rupali Ugrankar (UT Southwestern Medical Center).

### Triglyceride analysis

The Gal4 driver females were mated with UAS-microRNA males. The resulting F1 adult flies were collected, incubated for one week, and used for triglyceride (TG) analysis as described[8]. Briefly, multiple sets of six F1 flies of both sexes were collected separately and homogenized with lysis buffer (PBS supplemented with 0.05% SDS). The lysates were heat-inactivated for 30 minutes at 65°C and centrifuged to remove tissue debris (18,000g, 3min). The resulting supernatant was transferred into new tubes, mixed with Infinity solution (Thermo Scientific) in a 96-well plate, incubated for 5 minutes, and used to measure optical density (OD_500nm_).

### Developmental time analysis

Act5C-Gal4 driver females and UAS-miRNA males were placed in vials to mate for three days. The flies were then transferred to a new vial and kept for one day prior to collecting embryos. To measure the developmental time (DT), we counted newly-eclosed adult flies in the vial once a day until no new adults emerged. The DT of each miRNA line was estimated as the time elapsed from the embryo collection to the maximum adult emergence. The control, W^1118^ mated with the same Act5C-Gal4 driver, was run in parallel with each experimental batch, and the control DT was used to calculate relative DT of the miRNA lines in the same batch.

### RNA extractions and reverse-transcriptase qPCR

Total RNA was extracted using the TRIzol (Thermo Scientific) by the manufacturer’s instructions. To generate cDNA, 1μg of total RNA was reverse-transcribed with Moloney Murine Leukemia Virus Reverse Transcriptase (M-MLV RT) and random hexamers. Gene expression was analyzed with qPCR (Applied Biosystems) with SYBR green master mix reagent (Applied Biosystems) and specific primers (Table 1). The values for gene expression were normalized by expression of ribosomal protein 49 (Rp49), an endogenous control.

**Table 1:** Primer sequences for qPCR.

Primers	Sequences	Primers	Sequences
Rp49-F	CGATGTTGGGCATCAGATACT	RP49-R	TGCTAAGCTGTCGCACAAAT
APS-F	GAATGAGGCGGAGGTACTCTT	APS-R	CGGCTGTCACCGATGACTC
Atg5-F	CCGGAGCCTTTCTATCTGATGA	Atg5-R	CCTGGTGTTCGGCGCTTAT
Babo-F	CTACCAGATTATGTGCCACAC	Babo-R	TACTGGTGCCCGTGAAGCAA
Gr47b-F	ACAGCCTCCTGCTCTACTGG	Gr47b-R	GTCCACCTGTTTGAAAACGCA
RPL41-F	AAGTGGCGTAAGAAGCGTATG	RPL41-R	CCTTGCACGCATCTTTCTGC
Scamp-F	TGTGTAAAGCCGTGCTTCTAC	Scamp-R	GCCAACAACGTCATGGTGTAA
Ter94-F	AGTCGCGGTGTCCTTTTCTAC	Ter94-R	GGACCCTTGACTGAGATGAAGTT

## Results

### Screening for miRNAs controlling adiposity in *Drosophila melanogaster*

**Fig 1:**
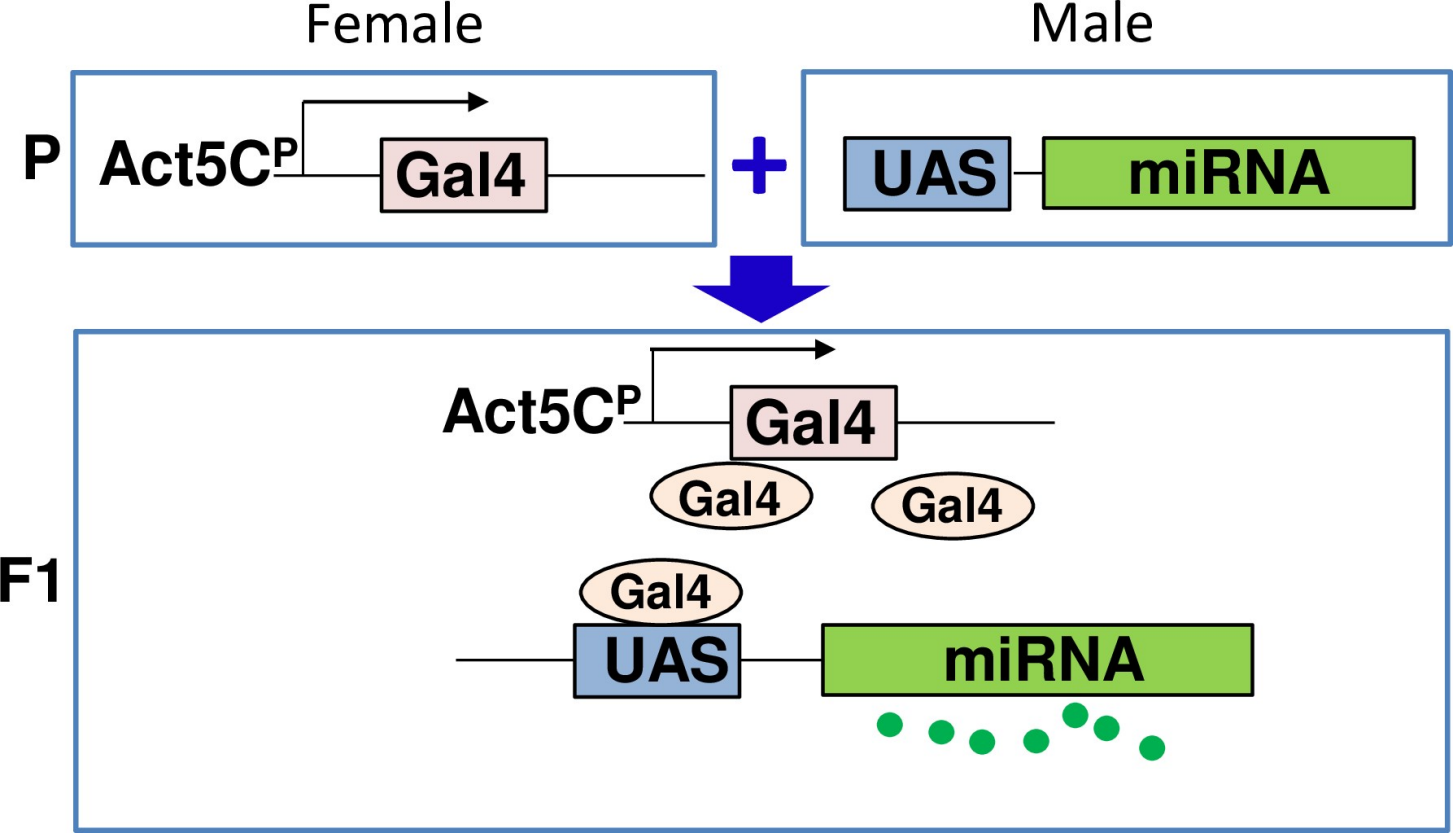
Experimental scheme of microRNA expression. The UAS/Gal4 binary transgene expression system was used to overexpress microRNAs (miRNA). Gal4 transactivator was expressed under the control of the Act5C promoter (Act5C^P^). Each miRNA was under the control of the upstream activating sequence (UAS) (UAS-miRNA). When female Act5C^P^-Gal4 was mated to male UAS-miRNA (Parental generation, P), both genetic components (UAS/Gal4) were combined in the filial generation 1 (F1) and ubiquitously produced the specific miRNA in all actin-producing cells.

**Fig 2:**
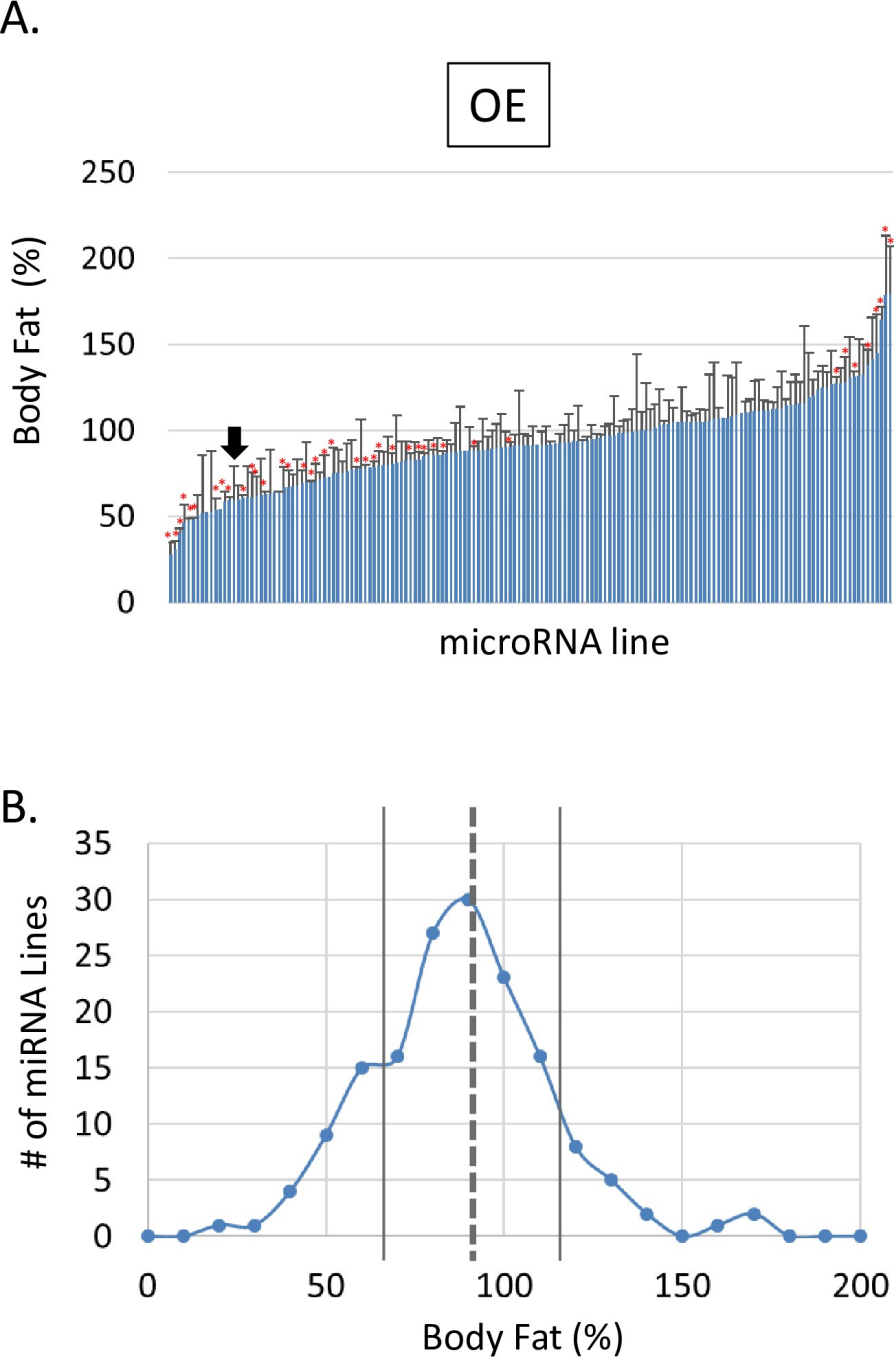
Gain-of-function miRNA lines altered body fat. **(A)** The amounts of triglyceride (TG) of F1 males from the cross of Act5C-Gal4 and UAS-miRNA were determined using the colorimetric method. Then, the TG content of each miRNA line was compared to that of the W^1118^ control male; the relative amount of body fat (%) was calculated using the following formula. Relative Body Fat (%) = (TG_miRNA_/TG_control_) X 100; *, P < 0.01 by Student’s t-test. Each bar graph represents a different miRNA line; error bars represent the standard deviation. The black arrow shows miR-969. **(B)** Each miRNA line was assigned to a bin depending on their male body fat contents. The bins were between 0% and 200% fat contents with 10% increments. The dotted line represents the mean (91.7%); two solid lines (66.5% and 116.9%) denote one standard deviation from the mean.

Using the yeast Gal4/ UAS binary transgene expression system, we screened for miRNAs which control the amount of body fat. We crossed 160 UAS-miRNA gain of function (GOF) lines which represent 101 different miRNAs, to the Act5C-Gal4 driver to achieve ubiquitous expression of the miRNAs (Fig 1). The resulting F1 adult flies were collected, sorted by sex, frozen, homogenized, and heat-treated. The subsequent homogenate was then used to determine triglyceride concentration, representative of the total body fat using colorimetric method[8]. We tested three to ten miRNA lines as one batch with controls generated by crossing W^1118^ to the same driver. For data analysis, we set the fat content of control males to 100% and determined the body fat of each miRNA-expressing line compared to its control in the same batch (Fig 2A). Noticeably, the mean fat content (FC) of all GOF miRNA lines was 91.7% (Fig 2B, S1 Table); the lowest and highest fat contents were 28% and 179%, respectively. The Bonferroni corrections are often used to reduce the number of false positive data when a large number of statistical tests are performed. Since we tested 160 miRNA lines, we analyzed the data with the Bonferroni corrections (S1 Table).

We selected 47 miRNA lines, whose FC were deviated by at least one standard deviation from the mean (FC < 66.5, or FC > 116.9). We further narrowed down our miRNA collection by selecting lines whose fat contents were consistently altered across both sexes; we named this final set microCATs (**micro**RNAs **C**ontrolling **A**dipose **T**issue) (Table 2). One of these, miR-33, was previously demonstrated to regulate both fatty acid metabolism, glucose metabolism, and insulin signaling [26,27].

**Table 2:** The microCATs.

Stock			TG: % of Control		SD	
	miR	Stock #	M	F	M	F
**1**	**miR-999**	**44123**	**28**	**65**	**6.6**	**22.1**
**2**	**miR-133**	**59880**	**51**	**104**	**11.9**	**37.7**
**3**	**miR-276a**	**59897**	**54**	**93**	**na**	**13.3**
**4**	**miR-969**	**60624**	**59**	**91**	**19.9**	**12.9**
**5**	**miR-980**	**60637**	**62**	**83**	**21.5**	**na**
**6**	**miR-279**	**41147**	**63**	**35**	**na**	**na**
**7**	**miR-1000**	**41201**	**66**	**149**	**12.3**	**18.9**
**8**	**miR-33**	**59871**	**128**	**271**	**8.3**	**8.0**
**9**	**miR-981**	**60638**	**141**	**320**	**24.7**	**2.1**
**10**	**miR-1013**	**41215**	**179**	**303**	**34.3**	**11.0**

### Developmental time is negatively correlated with the amount of body fat

**Fig 3:**
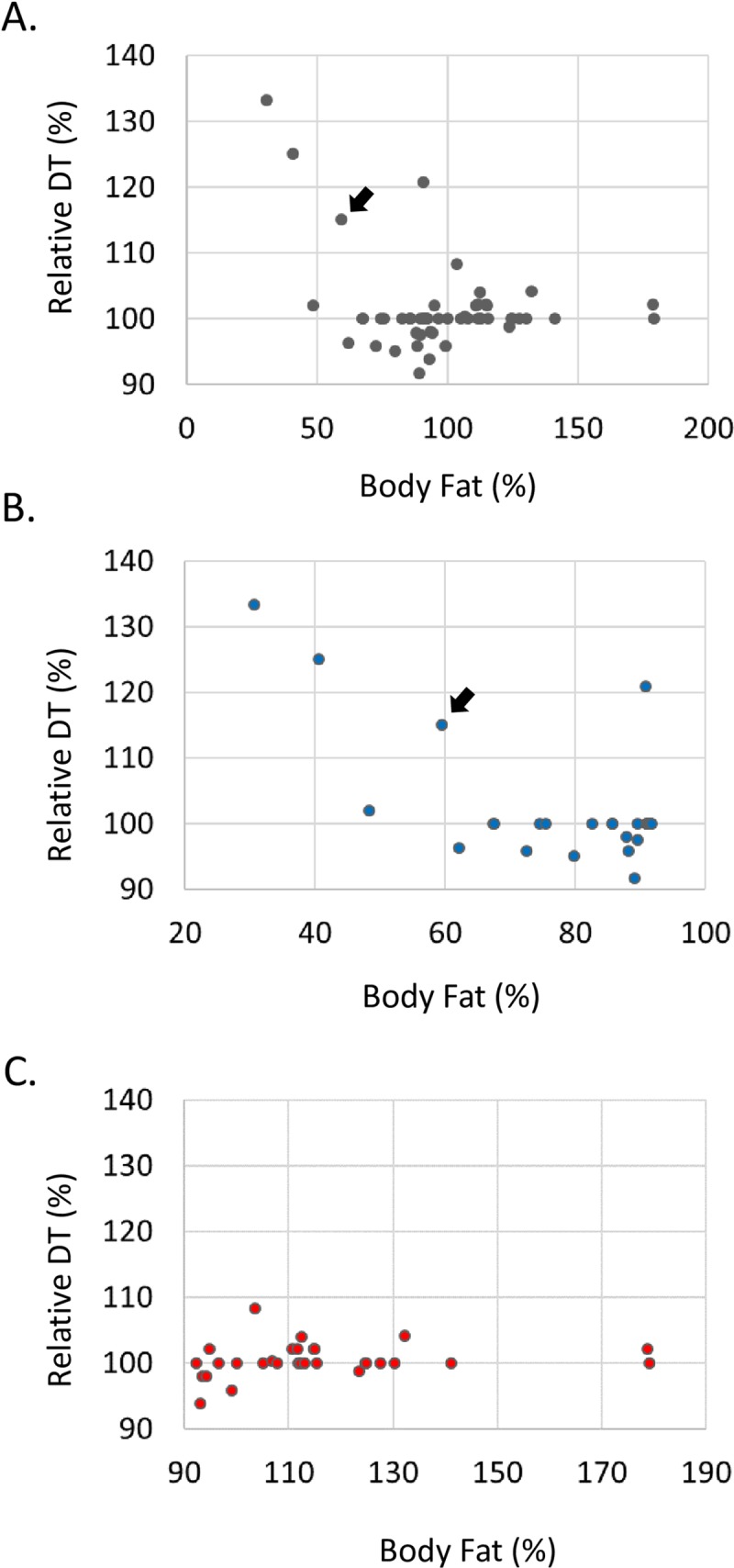
Negative correlation between the body fat content and developmental time. **(A)** Randomly-chosen 58 miRNA lines were used to plot egg-to-adult developmental time (DT) against male body fat content. Each coordinate represents the fat content and DT of a miRNA line. The linear correlation test in SPSS was used for a statistical test. Pearson correlation coefficient (r) was -0.330, and probability (P) value was 0.011. Relative DTs were calculated using the following formula: Relative DT (%) = (DT_miRNA_/DT_control_) X 100. Controls were generated by crossing W^1118^ to the same Act5C-Gal4 driver. The black arrow shows miR-969. **(B)** Only the 28 miRNA lines with low body fat were considered in this analysis. Pearson correlation coefficient (r) was -0.744, and P value was lower than 0.011. The black arrow shows miR-969. **(C)** The other 33 miRNA lines with high body fat were analyzed. Pearson correlation coefficient (r) was 0.206, and P value was 0.249.

We hypothesized that an animal with elevated energy storage (high fat content) would have accelerated development. To test this possibility, we measured egg-to-adult developmental time (DT) of 58 randomly-chosen miRNA lines whose fat contents had been previously determined (S2 Table). We plotted total fat content against DT of each miRNA line (Fig 3A). We found a weak but significant negative correlation between the fat contents and DTs by Pearson correlation test (r = - 0.330, p = 0.011). When we analyzed only the miRNA lines whose fat contents were below the mean of the total miRNA lines, we found a much stronger correlation between the fat contents and DTs (r = - 0.744, p< 0.001) (Fig 3B). However, when the other miRNA lines with high fat contents were analyzed, no correlation was found between the body fat contents and DTs (r = 0.206, p = 0.249) (Fig 3C).

### Loss of function screening to identify miRNAs controlling body fat

**Fig 4:**
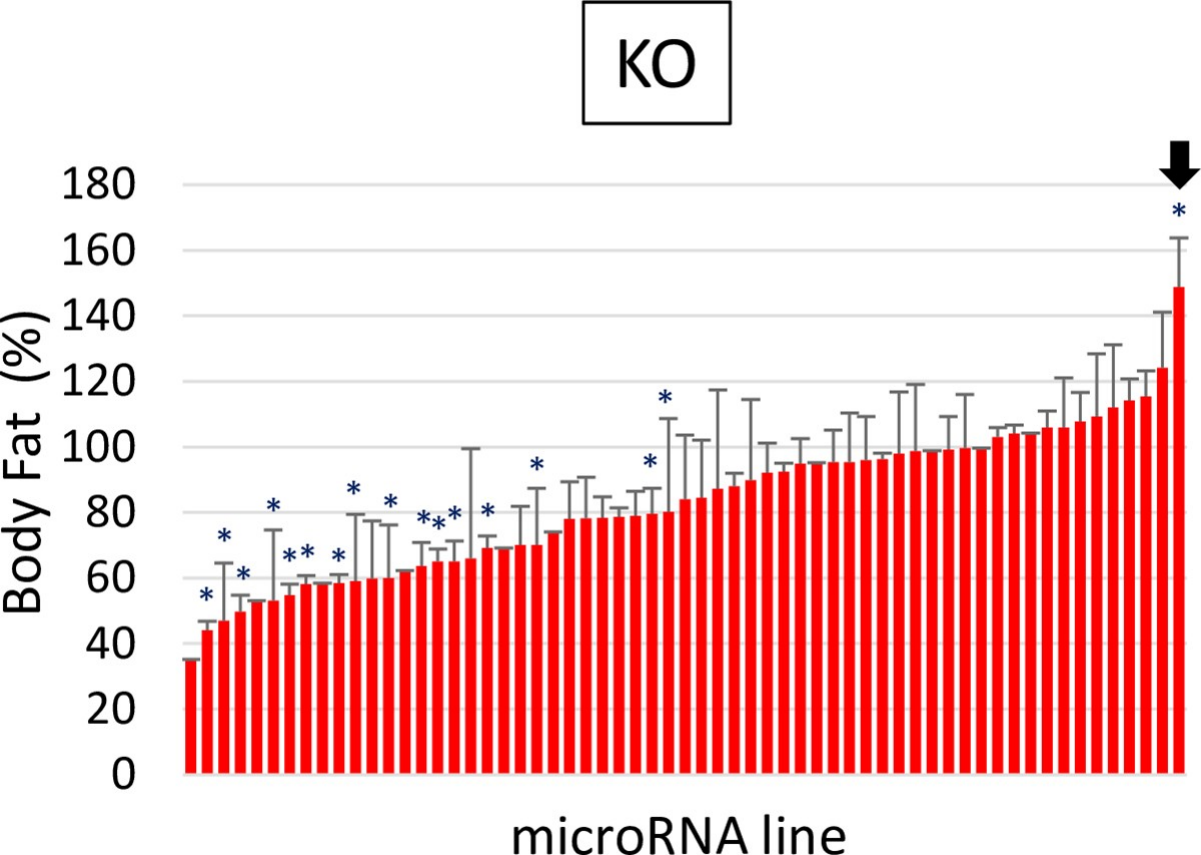
Loss-of-function miRNA heterozygotes altered body fat. MiRNA knock-out (KO) lines were mated with the Act5C-Gal4 driver. The resulting F1 heterozygotes were comparatively analyzed against control flies that were generated from the cross between W^1118^ and the same Act5C-Gal4 driver. The relative body fat of each miRNA line was calculated by normalizing each heterozygote’s triglyceride (TG) to the control TG. Relative Body Fat (%) = (TG_miRNA_/TG_control_) X 100, *: P < 0.01 by Student’s t-test. The black arrow shows miR-969.

To complement the GOF miRNA screening data (Fig 2), we further investigated the necessity of miRNAs using the miRNA knock-out (KO) library[28]. To overcome the homozygous lethal phenotypes of multiple KO lines, we generated F1 heterozygotes by mating flies from the loss-of-function (LOF) library with flies carrying the Act5C-Gal4 driver which had been used previously for the GOF miRNA screens. The resulting F1 flies were heterozygotes and, since the same driver was used, contained at least 50% identical genetic background as the F1 flies of the GOF screens. We analyzed 61 heterozygote lines (Fig 4) and found that the lowest body fat was 35% and the highest was 149% when compared to the control group (S3 Table). The average body fat of the miRNA heterozygotes was 82.9%, and their standard deviation was 22.4. We focused on the miRNA lines, whose fat contents deviated at least one standard deviation from the mean (FC < 60.4, or FC > 105.3). Among the focus group, we selected eight lines whose fat contents were similarly affected both in males and females (Table 3). Combining this miRNA heterozygote data with microCATs (Table 2), we found that miR-969 is a critical regulator to control body fat contents. Overexpression of miR-969 decreased the percentage of body fat; inversely, reduction of miR-969 increased body fat.

**Table 3:** Heterozygote microRNAs regulating body fat.

Stock			TG: % of Control		SD	
	miR	Stock #	M	F	M	F
**1**	**miR-263b**	**58903**	**47**	**120**	**17.6**	**30.7**
**2**	**miR-193**	**58898**	**50**	**93**	**5.1**	**5.9**
**3**	**miR-317**	**58926**	**55**	**124**	**3.4**	**7.1**
**4**	**miR-278**	**58909**	**58**	**110**	**2.6**	**12.8**
**5**	**miR-318**	**58927**	**106**	**265**	**5.0**	**8.6**
**6**	**miR-375**	**58931**	**106**	**256**	**15.1**	**9.3**
**7**	**miR-2b-1**	**58915**	**124**	**335**	**16.9**	**12.5**
**8**	**miR-969**	**58950**	**149**	**256**	**15.0**	**13.5**

### MiR-969 regulates Gr47b expression

**Fig 5:**
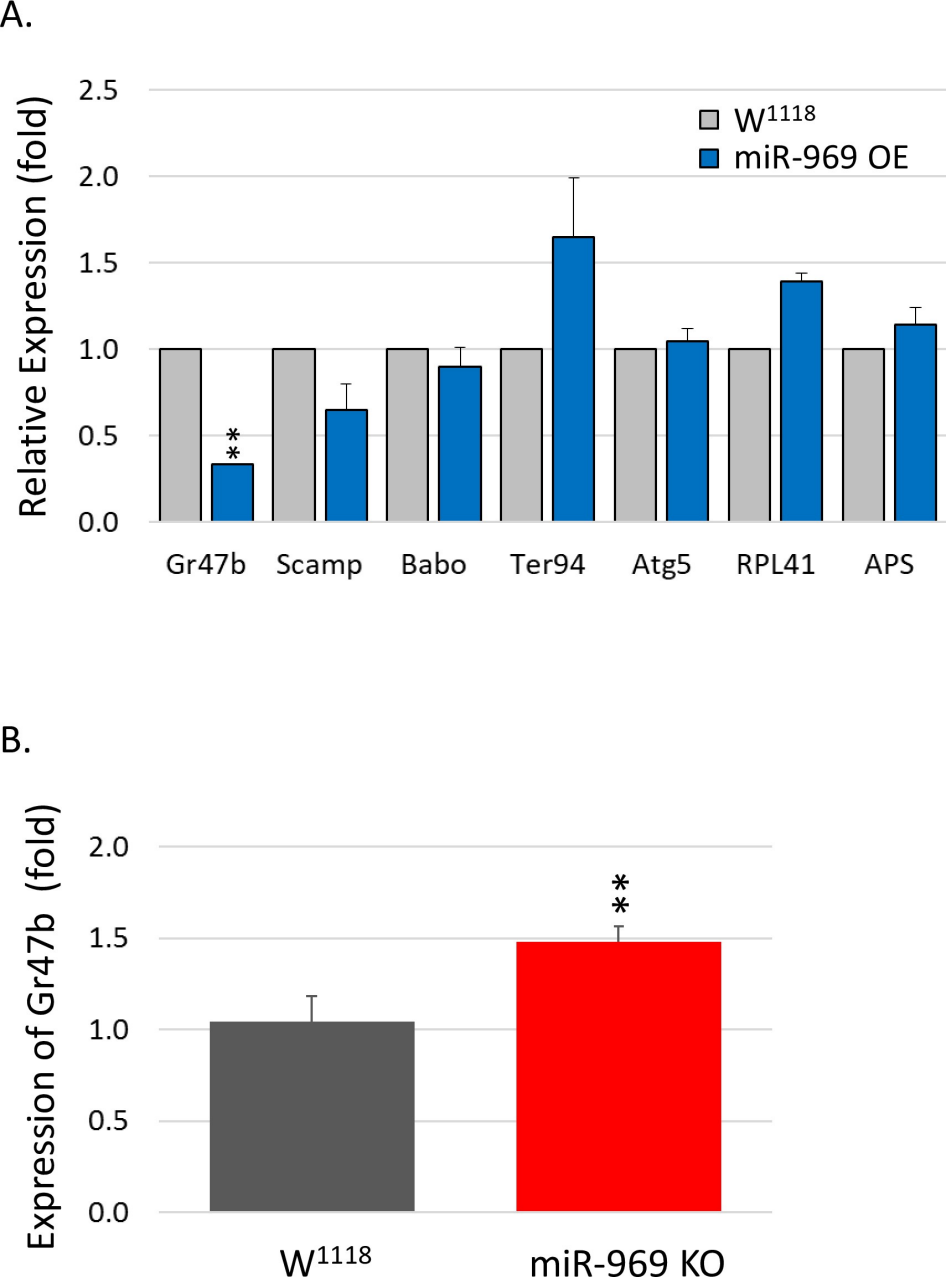
Altered expression of possible target genes of miR-969. **(A)** Overexpression of miR-969 reduced Gr47b expression. The miR-969 overexpression line was generated by crossing the UAS-miR-969 with the Act5C-Gal4 driver. Total RNA was extracted from the resulting F1 adults and converted to cDNA for qPCR analysis. The expression levels of the seven candidate genes were determined, and they were Gustatory receptor 47b (Gr47b), Secretory carrier membrane protein (Scamp), Activin receptor Baboon (Babo), Transitional endoplasmic reticulum ATPase (Ter94), Autophagy protein 5 (Atg5), Ribosomal protein L41 (RPL41), and Nudt3 *Drosophila* homolog (APS). **(B)** MiR-969 knock-out line increased Gr47b expression compared to the W^1118^ control. Gr47b expression was determined by qPCR. Statistical analysis was performed by Student’s T-test. *: P < 0.01.

To better understand the miR-969 function in adiposity control, we attempted to identify miR-969 target genes. First, we used two computational algorithms (TargetScanFly6.2 and miRanda-mirSVR) to identify target genes. The first algorithm (TargetScanFly6.2) identified 27 conserved targets; the other algorithm (miRanda-mirSVR) identified 869 targets. To narrow down putative target genes for qPCR verification, we used the following criteria: (1) common target genes in both algorithms, (2) known genes regulating metabolic pathways, (3) known genes regulating cell proliferation and differentiation, and (4) genes encoding receptor molecules or enzymes. With further extensive literature review, we selected seven candidate genes (Gr47b, Scamp, Babo, Ter94, Atg5, RPL41, and APS). All genes selected have 3’ UTR complementary sequences to miR-969 and likely modulate the amount of body fat content. APS was previously shown to regulate insulin signaling and fat content in both fruit flies and humans[29]. To experimentally confirm whether miR-969 altered expression of the candidate genes, we tested the expression of the seven candidate genes in miR-969 GOF flies using qPCR. Gustatory receptor 47b (Gr47b), was the only candidate gene whose expression was significantly reduced by overexpression of miR-969 (Fig 5A). To further confirm miR-969—Gr47b regulation, we measured Gr47b expression in miR-969 KO flies. As predicted, miR-969 KO significantly increased expression of Gr47b (Fig 5B).

UAS-microRNA sponge lines are valuable resources since expression of miRNA sponges can be used to reduce the miRNA expression in a tissue-specific manner[30]. Before testing tissue-specific effects of miR-969 expression, we first crossed UAS-miR-969 sponge line with Act5C-Gal4 ubiquitous driver to test whether the miR-969 sponge line can increase Gr47b. However, Gr47b expression was not significantly changed; nor was body fat increased in the Act5C-Gal4 > miR-969 sponge flies probably due to levels of miR-969 sponge expression (S1 Fig).

### Gr47b, a miR-969 target, regulates body fat

**Fig 6:**
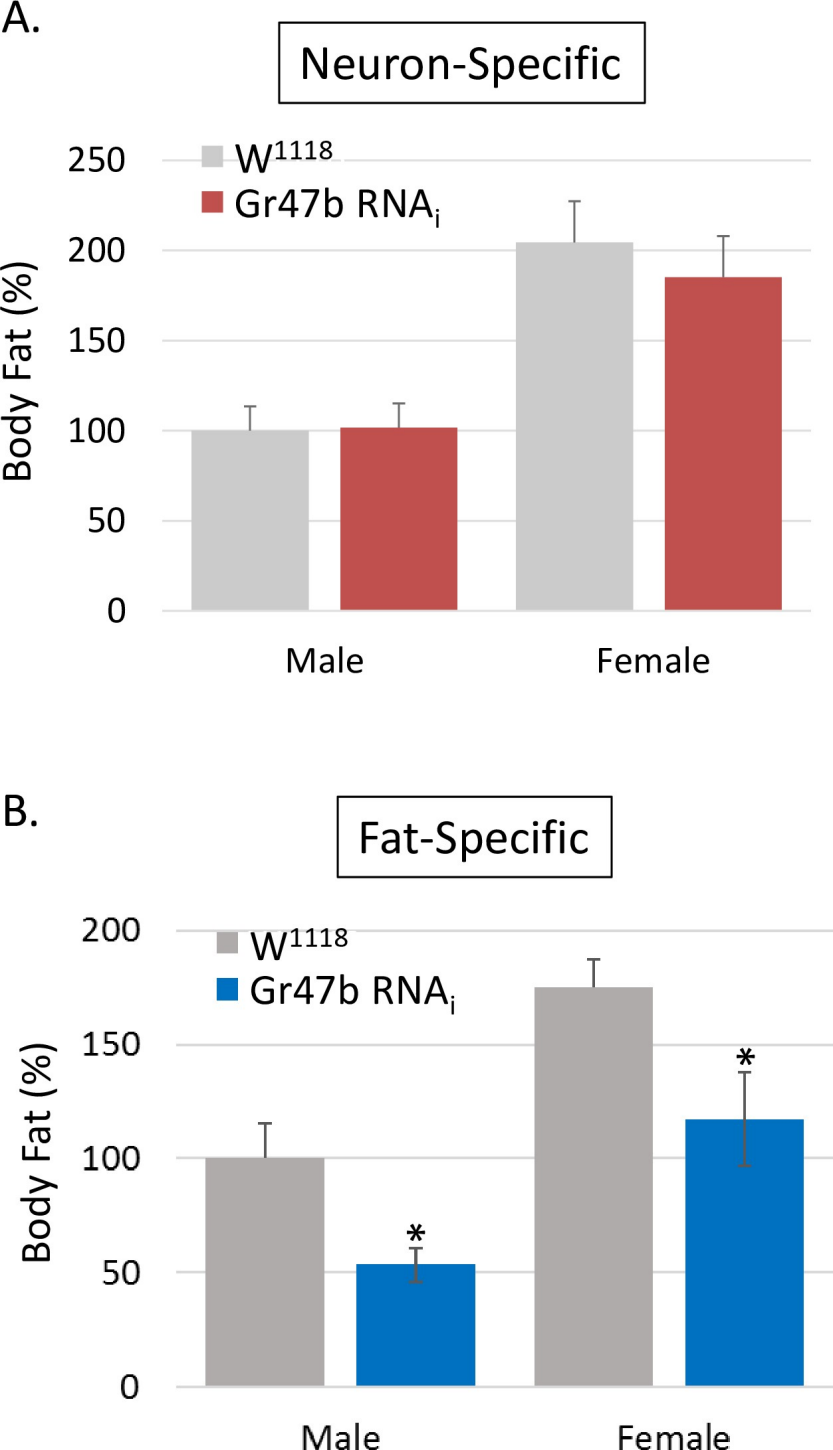
Adipose-specific knocking-down of Gr47b significantly reduced the amount of body fat. **(A)** Gr47b RNAi males were mated with nSyb-Gal4 females. The resulting F1 adults were collected, sorted by sex, homogenized, and used to determine body fat. The control adults were generated from the cross between W^1118^ and nSyb-Gal4. Relative Body Fat (%) = (TG_miRNA_/TG_control_) X 100. Error bars represent the standard deviation. **(B)** Gr47b RNAi males were mated with fat-specific Dcg-Gal4 females. The resulting F1 adults were analyzed together with their controls. Statistical analysis was performed by Student’s T-test. *: P < 0.01.

Considering the expression of Gr47b was significantly affected by miR-969, we hypothesized that Gr47b expression was responsible for the miR-969 adiposity phenotype. To test this, we knocked-down Gr47b using nSyb-Gal4, a neuron-specific driver since Gr47b was predicted to be a gustatory receptor[31,32]. We reasoned that neuron-specific knock-down of the gustatory receptor would alter taste sensation, thereby changing food intake and body fat. However, neuron-specific knocking-down of Gr47b did not affect body fat (Fig 6A). To test any possible functions of gustatory receptors (GRs) in adipose tissue, we knocked-down Gr47b in adipose-tissue specifically using Dcg-Gal4 driver[33]. Adipose-specific knocking-down of Gr47b significantly reduced body fat contents in both sexes (Fig 6B). However, knocking-down of Gr47b using another adipose-specific driver, Lsp2-Gal4 has not reduced body fat (S2 Fig). Since Dcg expression occurs earlier than Lsp2 expression during embryogenesis and larval development of *Drosophila melanogaster*[34,35], Gr47b might play a role in proliferation and differentiation of the adipocyte progenitors. Alternatively, this discrepancy might be due to different Gal4 expression of the two adipose-specific drivers. To further test the gustatory receptor functions in adipose tissues, we selected three more GR RNAi lines (Gr10b, Gr59e, and Gr59f) which are closely related to Gr47b[36]. When they were knocked-down in adipose tissues using Dcg-Gal4, two of the GRs (Gr10b and Gr59e) significantly reduced fat contents (S3 Fig). This data suggested that GRs played an important role in adipose tissue and controlled body fat contents; further, Gr47b mediated the effects of miR-969 on adiposity in *Drosophila melanogaster*.

## Discussion

The miRNA family consists of 1–2% of the total number of protein-coding genes in the fruit fly, mouse, and human[37]. Considering each miRNA regulates approximately 100 targets[38], miRNAs have tremendous regulatory potential in modulating gene expression post-transcriptionally. For instance, miRNAs in the human genome are predicted to regulate over 60% of the total protein-coding genes[10]. Thus, we performed a GOF screen using the UAS-miRNA library and identified miRNAs controlling body fat contents in both sexes in *Drosophila melanogaster*. To diminish minor but persistent fluctuations of experimental conditions during the extended periods of data collection and experimenters’ errors, we used relative values for the control of each batch. We reasoned that such experimental variations and errors would similarly affect both the experimental group and the control group in the same batch. Thus, it would minimize systemic errors among different batches when each set was combined into one complete data.

To reliably identify valid miRNAs controlling body fat, we selected the miRNAs which significantly alter body fat in both sexes and labeled them as microCATs (Table 2). We combined the microCAT data with the heterozygous KO mutant data (Table 3) and identified miR-969 as an essential fat regulator in fruit flies. MiR-969 was previously shown to control primordial germ cell numbers[39]; however, functions in adiposity and metabolism have not been addressed. We further identified Gr47b was a *bona fide* miR-969 target by assessing gene expression in miR-969-overexpressing flies. Gr47b is predicted as a member of the gustatory receptor family. However, Gr47b was expressed highest in the fat body among larval tissues as shown in the FlyAtlas (http://flyatlas.org/tissues.cgi)[40]; the larval fat body expressed Gr47b more than even the larval central nervous system where general gustatory receptor expression is expected to be the highest. This tissue-specific analysis of Gr47b expression suggests the idea that Gr47b might play a role in adipose tissue.

Insect gustatory receptors (GRs) detect nonvolatile compounds and regulate behavior preferences on food selection, mate choice, and egg deposition site selection[32,41]. In the *Drosophila melanogaster* genome, the GR family contains 68 members which share a conserved C-terminus motif[32,41]. Among the GRs, Gr5a and Gr64f are responsible for sweet taste[42]; Gr33a and Gr66a are responsible for bitter taste in sensory neurons[43]. Gr68a and Gr32a regulate courtship behavior and sexual preference[44]. The gustatory receptors were predicted to be G-protein-coupled receptors (GPCRs) as the mammalian chemosensory receptors[41]. Supporting the notion, GR-related olfactory receptors have been shown to be ligand-gated channels and GPCRs[45,46]. We first knocked-down Gr47b neuron-specifically, but the mutation did not affect fat contents. Sweet taste receptors were expressed in digestive tracts and regulated dietary sugar transport capacity, appetite, and insulin secretion in mice[47,48]. The sweet taste receptors were shown to be induced during adipocyte differentiation; further, the knockout mice of sweet taste receptors significantly reduced body weight and fat content[49–51]. To test possible functions of Gr47b in adipose tissue, we knocked-down Gr47b fat-specifically, which significantly reduced body fat and recapitulated miR-969 GOF phenotype (Fig 6). This data strongly suggest that Gr47b might work as a nutrient sensor in adipose tissue to control lipid metabolism, adipocyte differentiation, and tissue remodeling in fruit flies. However, it is worth noting that miR-969 is not conserved in humans, and there is no human Gr47b ortholog. Thus, the functions of miR-969 and its target, Gr47b might be limited to insects.

Developmental processes such as cell migration, tissue patterning, and assembly of functional tissues are tightly regulated to achieve precise individual organism architect. Hippo, insulin, and ecdysone signaling pathways are well understood to control the body size and growth rate in fruit flies [52–54]. Likewise, environmental cues such as food availability, temperature, and amount of daylight affect insect developmental time (DT) [55–57]. The founding members of microRNAs, lin4 and let7, were originally identified as regulators of developmental timing in *C*. *elegans*[58–60]. We attempted to test the possibility whether the amount of stored body fat (accessible energy) regulated DT. Through combining the miRNA GOF data and DTs, we demonstrated body fat contents negatively correlated with DTs (Fig 3). Noticeably, the subset with low body fat had a stronger correlation to DT than the whole set; however, the other subset with high body fat contents showed no correlation. A simple explanation of this data would be that miRNA GOF mutations caused the flies to become unhealthy, which made the mutants spend more time searching out and digesting foods, thereby triggering an extension of DT. However, we observed most of the low-fat mutants seemed normal and were able to reproduce. Thus, we are tempted to propose a ‘fat mass checkpoint’ hypothesis. An organism must accumulate a critical amount of energy to advance to the next developmental stage. Thus, significant reduction of body fat may cause overall developmental process to be delayed until the organism accumulates critical amount of energy (fat deposition) to pass the ‘fat mass checkpoint’. However, we did not observe increased amounts of fat accelerating an organism’s developmental process, which suggested the fat mass checkpoint may be a required condition to advance the developmental program, but not a sufficient factor. Since fat cells (adipocytes) actively respond to and regulate the metabolic state [61–63], we predict some adipokine(s) may work as regulator(s) to communicate with other endocrine glands, such as the steroidogenic prothoracic gland for ecdysone, to overcome ‘fat mass check point’ and continue the developmental program.

## Supporting information

S1 Fig Ubiquitous expression of miR-969 sponge did not alter body fat nor Gr47b expression. **(A)** Act5C > miR-969 sponge (SP) line was generated by crossing the UAS-miR-969 SP with the Act5C-Gal4 driver. Total RNA was extracted from the resulting F1 adults and converted to cDNA for qPCR analysis. The expression level of gustatory receptor 47b (Gr47b) was determined. **(B)** miR-969 SP males were mated with Act5C-Gal4 females. The resulting F1 adults were collected, sorted by sex, homogenized, and used to determine body fat. The control adults were generated from the cross between W^1118^ and Act5C-Gal4. Relative Body Fat (%) = (TG_miRNA_/TG_control_) X 100. Error bars represent the standard deviation.
(TIF)

S2 Fig Lsp2-Gal4 > Gr47b RNAi did not reduce body fat. UAS-Gr47b RNAi males were mated with Lsp2-Gal4 females. The resulting F1 adult males were collected, homogenized, and used to determine body fat. The controls were generated from the cross between Lsp2-Gal4 and W^1118^, and UAS-Gr47b RNAi and W^1118^. Error bars represent the standard deviation.
(TIF)

S3 Fig Adipose-specific knocking-down of multiple gustatory receptors significantly reduced the amount of body fat. UAS-Gr10b RNAi, UAS-Gr59e RNAi, and UAS-Gr59f RNAi males were mated with Dcg-Gal4 females. The resulting F1 adult males were collected, homogenized, and used to determine body fat. The control was generated from the cross between Dcg-Gal4 and W^1118^. Error bars represent the standard deviation. Statistical analysis was performed by Student’s T-test. *: P < 0.05.
(TIF)

S1 Table Body fat content in gain-of-function microRNA screen. (PDF)

S2 Table Body fat content vs developmental time in ubiquitously overexpressed microRNAs. (PDF)

S3 Table Body fat content in loss-of-function microRNA screen. (PDF)
