# Supplementary figures and images for: Screening of microRNAs controlling body fat in Drosophila melanogaster and identification of miR-969 and its target, Gr47b
Source: PLoS One. 2019 Jul 18;14(7):e0219707. doi: 10.1371/journal.pone.0219707 (PMC6638924; doi:10.1371/journal.pone.0219707)

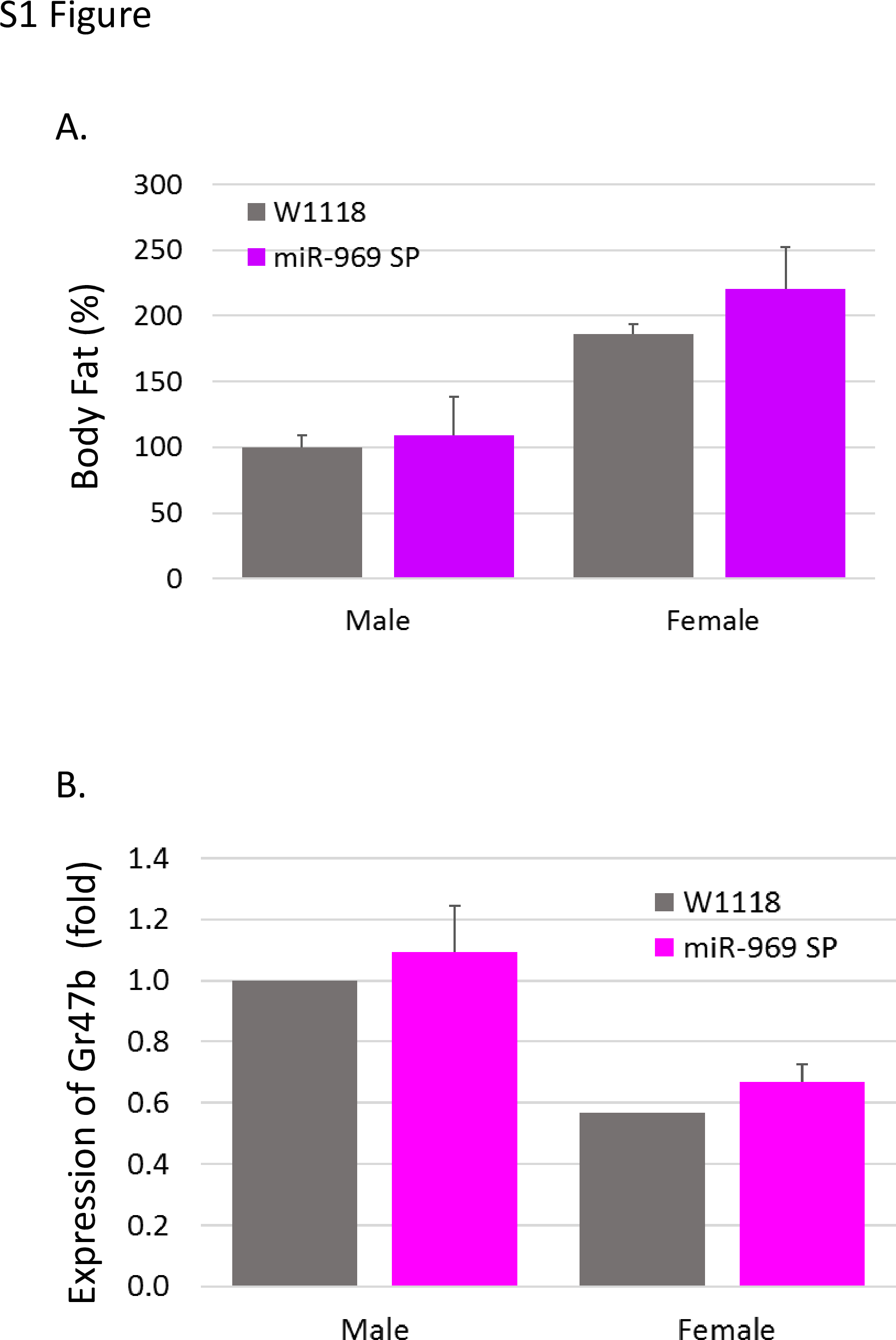

Supplement: S1 Fig — (A) Act5C > miR-969 sponge (SP) line was generated by crossing the UAS-miR-969 SP with the Act5C-Gal4 driver. Total RNA was extracted from the resulting F1 adults and converted to cDNA for qPCR analysis. The expression level of gustatory receptor 47b (Gr47b) was determined. (B) miR-969 SP males were mated with Act5C-Gal4 females. The resulting F1 adults were collected, sorted by sex, homogenized, and used to determine body fat. The control adults were generated from the cross between W1118 and Act5C-Gal4. Relative Body Fat (%) = (TGmiRNA/TGcontrol) X 100. Error bars represent the standard deviation. (TIF) [file pone.0219707.s001.tif]

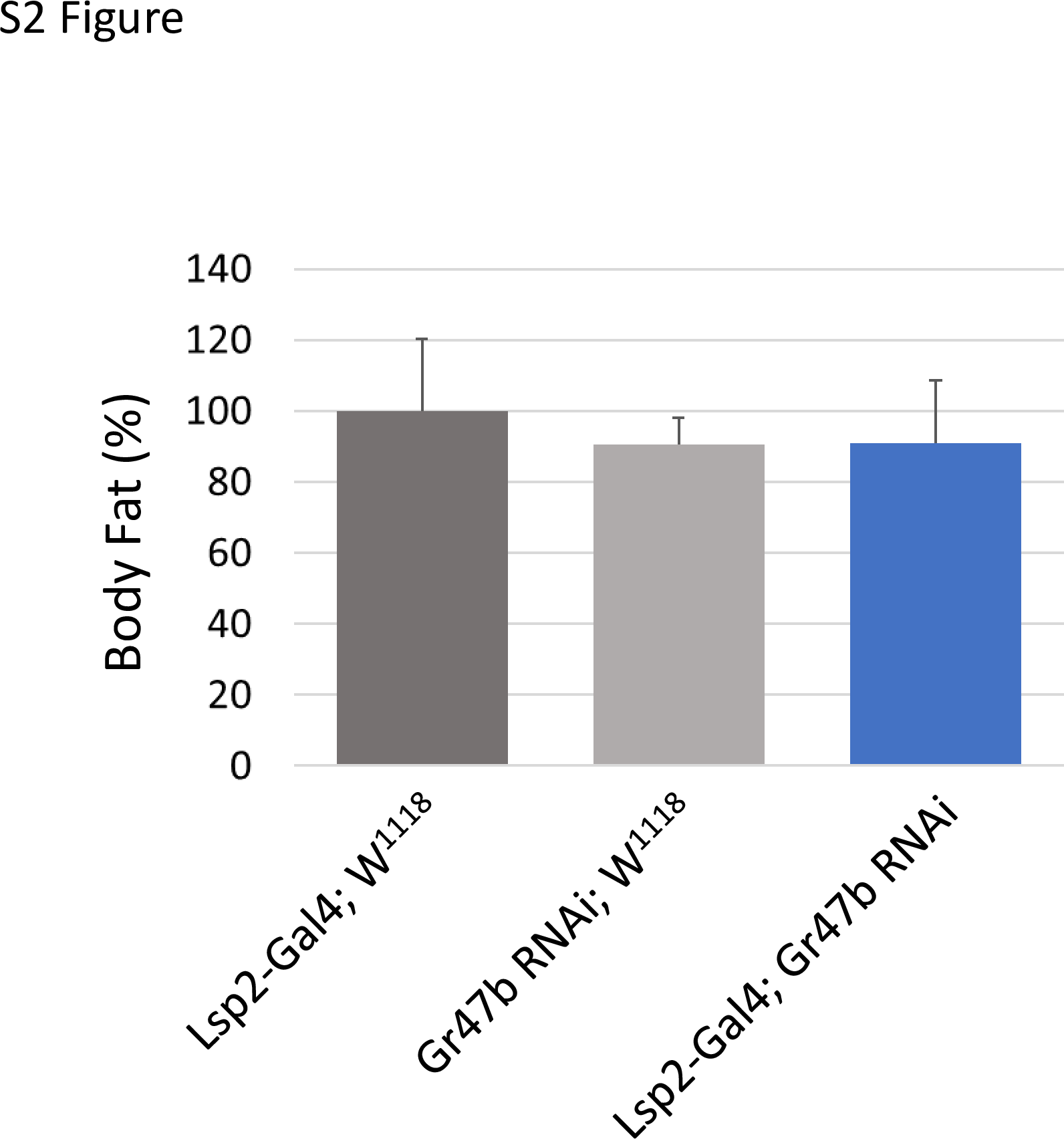

Supplement: S2 Fig — UAS-Gr47b RNAi males were mated with Lsp2-Gal4 females. The resulting F1 adult males were collected, homogenized, and used to determine body fat. The controls were generated from the cross between Lsp2-Gal4 and W1118, and UAS-Gr47b RNAi and W1118. Error bars represent the standard deviation. (TIF) [file pone.0219707.s002.tif]

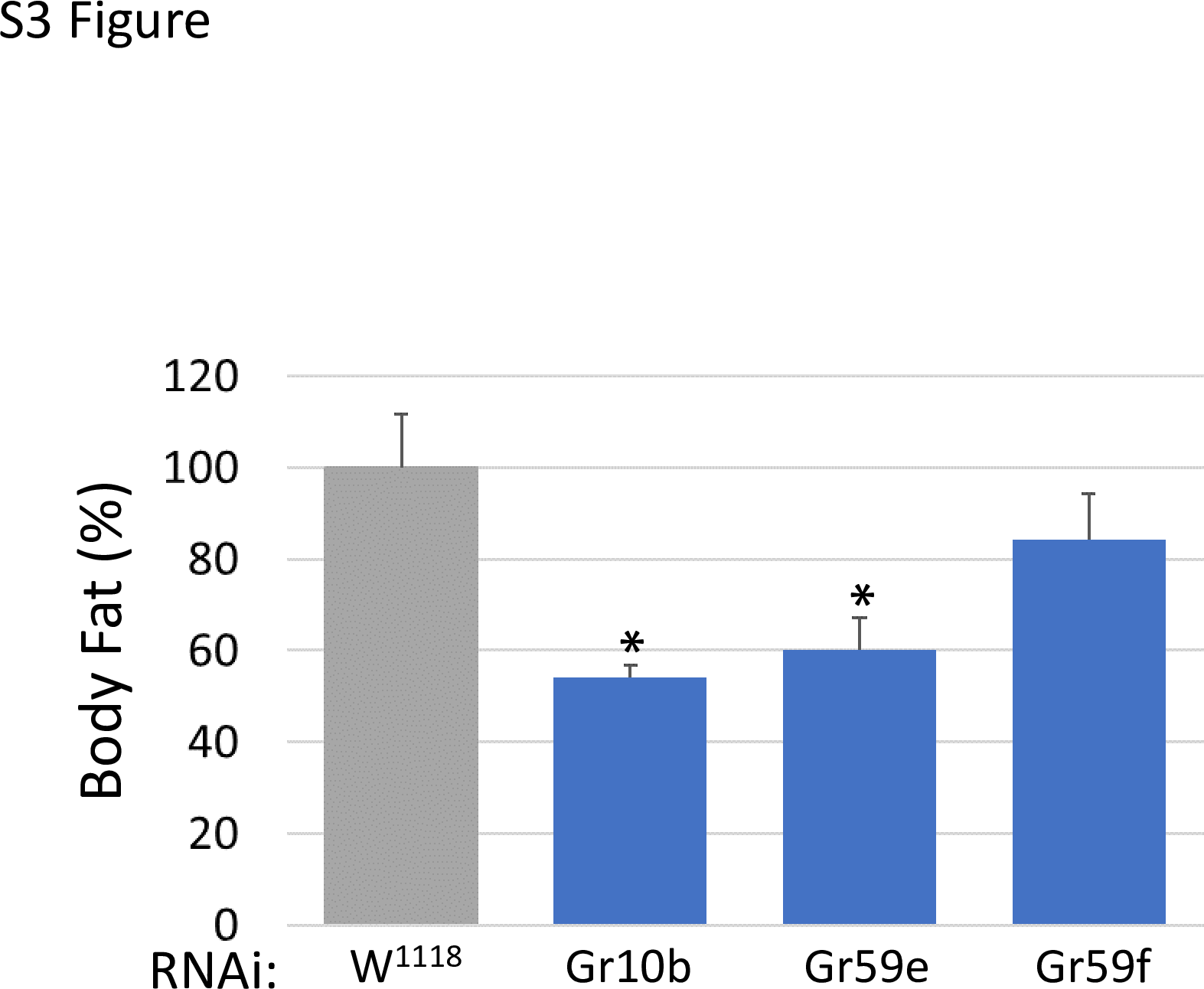

Supplement: S3 Fig — UAS-Gr10b RNAi, UAS-Gr59e RNAi, and UAS-Gr59f RNAi males were mated with Dcg-Gal4 females. The resulting F1 adult males were collected, homogenized, and used to determine body fat. The control was generated from the cross between Dcg-Gal4 and W1118. Error bars represent the standard deviation. Statistical analysis was performed by Student’s T-test. *: P < 0.05. (TIF) [file pone.0219707.s003.tif]
