# Supplementary material for: Screening of microRNAs controlling body fat in Drosophila melanogaster and identification of miR-969 and its target, Gr47b
Source: PLoS One. 2019 Jul 18;14(7):e0219707. doi: 10.1371/journal.pone.0219707 (PMC6638924; doi:10.1371/journal.pone.0219707)
Supplement: S1 Table — (PDF) [file pone.0219707.s004.pdf]

Supplement Table 1

Stock			TG: % to control		SD		T-test	
	miR	Stock #	M	F	M	F	M	F
1	mir-999	44123	28	65	6.6	22.1	*0.000	0.008
2	mir-100	41166	31	147	4.7	25.4	0.005	0.154
3	let-7	41171	41	141	2.2	29.9	0.008	0.193
4	mir-996	60654	46	233	10.4	61.3	0.011	0.221
5	mir-9a	41138	47	134	1.3	12.7	*0.000	0.018
6	mir-190	59884	48	116	0.7	12.6	0.007	0.741
7	mir-133	59880	51	104	11.9	37.7	0.078	0.139
8	mir-219	59890	52	114	34.0	32.0	na	na
9	mir-252	59891	52	176	na	na	na	na
10	mir-995	41199	52	169	36.2	38.9	0.141	0.959
11	mir-276a	41143	53	114	7.2	21.0	0.032	0.523
12	mir-276a	59897	54	93	na	13.3	0.021	26.200
13	bft	59894	58	134	5.9	39.5	0.032	0.202
14	mir-133	41132	59	145	1.9	21.3	0.022	0.148
15	mir-969	60624	59	91	19.9	12.9	0.073	0.028
16	mir-2b-1	59852	60	251	8.2	46.4	0.148	0.190
17	mir-984	41224	61	118	1.1	6.6	0.025	0.002
18	mir-307a	42026	61	139	18.0	36.9	0.071	0.691
19	mir-995	60651	61	130	14.6	6.8	0.025	0.002
20	mir-284	59904	62	133	11.4	4.1	0.050	0.761
21	mir-980	60637	62	83	21.5	na	0.094	na
22	mir-966	60621	63	144	1.3	7.2	*0.000	0.277
23	mir-274	41172	63	120	25.5	39.4	0.239	0.184
24	mir-279	41147	63	35	na	na	na	na
25	mir-303	59905	64	189	0.7	25.5	0.213	0.339
26	mir-1000	41201	66	149	12.3	18.9	0.048	0.305
27	mir-252	41127	67	152	9.7	5.4	0.022	0.097
28	mir-190	59885	67	137	6.9	26.9	0.099	0.210
29	mir-1000	60656	68	152	15.4	30.1	0.097	0.236
30	mir-967	60623	69	125	7.4	3.9	0.028	0.002
31	mir-286	60595	70	146	23.0	13.0	0.229	0.554
32	mir-2a-1	59850	70	449	0.5	3.3	0.024	0.012
33	mir-210	41179	71	104	9.6	13.7	0.032	0.002
34	mir-92a	59875	72	217	3.8	24.4	0.151	0.685
35	mir-992	41130	73	173	12.9	12.3	0.026	0.004
36	mir-929	60601	73	177	na	23.9	na	0.070
37	mir-10	41169	75	156	15.0	15.1	0.161	0.193
38	mir-306	59860	75	134	13.2	1.5	0.498	0.006
39	mir-971	60628	76	202	6.4	3.4	0.058	0.001
40	mir-1014	60668	76	146	16.2	8.2	0.081	0.848
41	mir-14	41178	77	169	17.2	19.2	0.101	0.421
42	mir-965	60619	77	125	1.4	11.7	0.005	0.096
43	mir-305	41152	78	450	28.2	122.8	0.386	0.699

44	mir-963	60617	78	124	1.6	6.7	0.049	0.007
45	mir-275	60596	78	131	na	na	na	na
46	mir-959	60613	79	152	3.0	10.9	0.018	0.016
47	mir-964	41148	80	158	8.6	27.2	0.048	0.121
48	mir-284	59903	80	101	7.7	26.3	0.097	0.218
49	mir-303	41193	80	137	16.4	4.5	0.482	0.002
50	mir-993	60648	81	126	6.0	2.9	0.036	0.103
51	mir-929	41184	81	222	27.4	9.0	0.833	0.392
52	mir-1010	60664	82	168	12.1	9.5	0.087	0.411
53	mir-1003	60659	83	191	11.1	15.4	0.092	0.448
54	mir-966	41211	83	114	3.8	3.6	0.008	0.020
55	mir-963	41216	83	146	10.1	12.6	0.096	0.012
56	mir-958	60612	83	161	4.4	26.4	0.018	0.238
57	mir-959	60614	85	111	2.0	2.6	0.004	0.016
58	mir-1000	60655	86	134	8.6	32.5	0.334	0.108
59	mir-959	60615	86	156	3.1	4.8	0.010	0.853
60	mir-989	41219	86	177	9.8	26.5	0.090	0.024
61	mir-994	41198	86	156	2.3	22.8	0.011	0.006
62	mir-956	60608	86	201	7.5	6.6	0.438	0.073
63	mir-278	41180	87	152	4.8	15.0	0.053	0.032
64	mir-10	59863	88	130	16.4	5.8	0.412	0.026
65	mir-927	60600	88	239	25.9	20.7	0.584	0.454
66	mir-2a-1	59849	88	434	na	29.4	na	0.549
67	mir-975	60634	88	270	13.7	41.8	0.365	0.589
68	mir-276b	60597	88	156	2.6	16.1	0.036	0.086
69	mir-986	41218	88	208	5.5	15.3	0.052	0.062
70	bft	41133	88	296	18.6	na	0.511	na
71	ban	60672	89	189	7.1	na	0.295	na
72	mir-1010	60663	89	222	10.6	5.0	0.552	0.034
73	mir-190	41154	89	222	14.3	64.5	0.619	0.972
74	mir-987	60643	90	114	19.6	31.6	0.499	0.415
75	mir-990	60647	90	190	9.6	32.9	0.174	0.074
76	mir-962	60616	91	114	2.5	7.5	0.030	0.008
77	mir-988	41196	91	218	6.6	26.4	0.128	0.286
78	mir-1012	41214	91	178	32.1	27.6	0.726	0.908
79	mir-973	41190	91	207	6.8	6.7	0.395	0.001
80	mir-994	60649	91	238	5.9	15.4	0.118	0.858
81	mir-988	60644	91	218	10.8	44.8	0.276	0.467
82	mir-276b	41162	92	138	na	na	na	na
83	mir-983-1	41194	92	234	7.3	3.4	0.502	0.005
84	mir-985	41213	92	204	11.0	14.9	0.532	0.001
85	mir-980	41191	92	254	2.1	24.9	0.592	0.018
86	mir-2b-1	59851	92	439	na	7.4	na	0.273
87	mir-975	60633	92	311	5.4	20.8	0.458	0.151
88	mir-275	59896	93	117	9.9	23.7	0.554	0.464
89	mir-965	60607	93	146	15.9	10.5	0.568	0.009
90	mir-975	60635	94	219	6.2	9.9	0.536	0.003

91	mir-10	59862	94	173	20.6	29.1	0.717	0.752
92	bft	59893	94	236	0.4	10.8	0.440	0.093
93	mir-974	41225	94	241	1.9	1.9	0.652	0.030
94	mir-318	59914	95	224	7.8	13.8	0.082	0.122
95	mir-281-1	59901	95	130	3.1	14.9	0.071	0.078
96	mir-958	41222	96	180	2.1	12.6	0.453	0.297
97	mir-986	60641	97	180	5.8	43.3	0.490	0.085
98	mir-284	41134	97	103	6.7	3.6	0.478	0.016
99	mir-210	59888	97	122	22.8	11.8	0.260	0.240
100	mir-957	60610	98	212	4.1	1.9	0.917	0.337
101	mir-10	59900	98	170	6.9	27.0	0.832	0.859
102	mir-987	41195	99	158	7.1	8.2	0.841	0.047
103	mir-1011	60666	99	183	13.3	28.7	0.962	0.774
104	mir-311	41163	100	177	44.6	32.3	0.984	0.815
105	mir-14	59868	100	185	10.2	28.2	0.975	0.936
106	mir-982	41192	100	206	27.5	21.3	0.987	0.002
107	mir-285	41160	100	130	12.5	4.4	0.974	0.564
108	mir-193	59887	101	152	13.9	24.4	0.896	0.940
109	mir-312	41144	103	196	20.7	26.5	0.819	0.537
110	mir-1007	41221	104	170	1.9	16.6	0.735	0.870
111	mir-1001	41202	104	160	13.5	34.2	0.812	0.387
112	mir-280	41164	105	132	8.3	28.7	0.390	0.962
113	mir-2b-2	59853	105	487	na	na	na	na
114	mir-306	59861	105	181	20.4	55.1	0.871	0.801
115	mir-961	41188	105	149	6.2	6.5	0.450	0.620
116	mir-976	41149	105	141	3.7	2.3	0.303	0.706
117	mir-282	52663	105	158	4.8	3.6	0.158	0.007
118	mir-1003	41220	105	306	7.6	43.4	0.472	0.079
119	mir-932	60604	105	166	6.7	21.3	0.529	0.278
120	mir-304	41170	105	124	27.1	8.4	0.861	0.006
121	mir-993	41197	106	166	33.0	9.8	0.074	0.050
122	mir-970	41189	107	262	6.3	3.0	na	na
123	mir-307a	59907	107	234	na	na	N	na
124	mir-1002	60657	108	151	24.0	14.3	0.712	0.312
125	mir-983-1	41217	108	187	22.6	11.3	0.686	*0.000
126	mir-11	59864	109	143	30.0	13.8	0.648	0.044
127	mir-2b-2	59855	110	455	na	29.4	na	0.182
128	mir-282	59902	110	136	6.5	4.8	0.051	0.636
129	mir-34	41158	111	223	7.5	15.9	0.584	0.141
130	mir-193	59886	111	195	17.9	29.4	0.384	0.218
131	mir-309	41181	111	162	18.3	29.9	0.388	0.416
132	mir-1011	41210	112	378	5.5	83.2	0.212	0.519
133	mir-1011	60665	112	204	4.9	38.4	0.501	0.315
134	mir-981	60639	112	269	4.0	40.5	0.350	0.118
135	mir-13b-2	59867	112	137	12.3	2.8	0.379	0.020
136	mir-1006	60661	113	198	21.3	13.7	0.551	0.248
137	mir-1	41125	114	205	3.7	26.0	0.173	0.062

138	mir-1014	60667	115	281	13.0	39.6	0.231	0.183
139	mir-972	60630	115	247	17.6	12.4	0.310	0.029
140	mir-314	59911	115	219	12.3	16.0	0.461	0.186
141	mir-276b	59899	116	100	44.8	21.3	0.593	0.189
142	mir-375	59916	119	211	26.2	6.7	0.318	na
143	mir-306	41156	120	168	9.5	14.8	0.073	0.028
144	mir-966	60620	124	156	10.6	32.7	0.086	0.504
145	mir-1009	41205	125	232	12.9	19.7	0.086	0.054
146	mir-1003	60658	125	154	8.8	14.0	0.132	0.160
147	mir-87	59873	127	237	18.9	6.7	0.094	na
148	mir-994	60650	127	193	4.1	24.9	0.011	0.034
149	mir-33	59871	128	271	8.3	8.0	0.206	0.006
150	mir-375	59917	128	169	14.3	16.9	0.049	na
151	mir-79	41145	130	222	23.9	28.5	0.246	0.234
152	mir-375	41182	131	169	3.4	30.2	0.008	na
153	mir-33	59872	132	197	21.1	8.8	0.074	na
154	mir-8	41176	132	219	17.6	19.9	0.188	0.212
155	mir-31b	41129	138	207	9.0	45.0	0.008	na
156	mir-981	60638	141	320	24.7	2.1	0.087	0.905
157	mir-13b-2	59866	145	257	22.6	43.8	0.031	0.027
158	mir-4966	41223	164	213	7.9	15.3	0.001	na
159	mir-1013	41215	179	303	34.3	11.0	0.020	0.092
160	mir-1006	60660	179	255	27.4	31.4	0.005	0.034

Asterisk (*) indicates statistical significance with the Bonferroni correction. (P < 0.00031)
