# Supplementary material for: Screening of microRNAs controlling body fat in Drosophila melanogaster and identification of miR-969 and its target, Gr47b
Source: PLoS One. 2019 Jul 18;14(7):e0219707. doi: 10.1371/journal.pone.0219707 (PMC6638924; doi:10.1371/journal.pone.0219707)
Supplement: S2 Table — (PDF) [file pone.0219707.s005.pdf]

Supplement Table 2

	miR	Stock #	TG	DT
1	mir-100	41166	30.7	133.3
2	let-7	41171	40.6	125.0
3	mir-190	59884	48.3	102.0
4	mir-969	60624	59.4	115.0
5	mir-980	60637	62.1	96.3
6	mir-190	59885	67.3	100.0
7	mir-1000	60656	67.6	100.0
8	mir-992	41130	72.5	95.8
9	mir-10	41169	74.5	100.0
10	mir-971	60628	75.5	100.0
11	mir-284	59903	79.7	95.1
12	mir-1003	60659	82.6	100.0
13	mir-1000	60655	85.6	100.0
14	mir-989	41219	85.7	100.0
15	mir-994	41198	85.7	100.0
16	mir-975	60634	87.9	97.9
17	mir-986	41218	88.2	95.8
18	mir-1010	60663	89.1	91.7
19	mir-987	60643	89.6	97.6
20	mir-990	60647	89.6	100.0
21	mir-988	41196	90.8	120.8
22	mir-1012	41214	90.9	100.0
23	mir-973	41190	90.9	100.0
24	mir-994	60649	91.1	100.0
25	mir-988	60644	91.3	100.0
26	mir-983-1	41194	91.6	100.0
27	mir-985	41213	91.6	100.0
28	mir-980	41191	91.7	100.0
29	mir-975	60633	92.3	100.0
30	mir-965	60607	93.1	93.9
31	mir-975	60635	93.5	98.0
32	mir-974	41225	94.2	97.9
33	mir-318	59914	94.9	102.1
34	mir-986	60641	96.6	100.0
35	mir-1011	60666	99.1	95.8
36	mir-982	41192	100.0	100.0
37	mir-1001	41202	103.6	108.3
38	mir-1003	41220	105.0	100.0
39	mir-970	41189	106.8	100.3
40	mir-983-1	41217	107.8	100.0
41	mir-34	41158	110.7	102.1
42	mir-1011	41210	111.6	102.2
43	mir-1011	60665	111.8	100.0
44	mir-981	60639	112.3	100.0

45	mir-13b-2	59867	112.4	104.0
46	mir-1006	60661	113.0	100.0
47	mir-1014	60667	114.7	102.2
48	mir-972	60630	115.0	102.1
49	mir-314	59911	115.4	100.0
50	mir-966	60620	123.5	98.8
51	mir-1009	41205	124.6	100.0
52	mir-1003	60658	124.8	100.0
53	mir-33	59871	127.6	100.0
54	mir-79	41145	130.3	100.0
55	mir-8	41176	132.3	104.2
56	mir-981	60638	141.1	100.0
57	mir-1013	41215	178.7	102.2
58	mir-1006	60660	179.2	100.0
