# Supplementary material for: Screening of microRNAs controlling body fat in Drosophila melanogaster and identification of miR-969 and its target, Gr47b
Source: PLoS One. 2019 Jul 18;14(7):e0219707. doi: 10.1371/journal.pone.0219707 (PMC6638924; doi:10.1371/journal.pone.0219707)
Supplement: S3 Table — (PDF) [file pone.0219707.s006.pdf]

Supplement Table 3

Stock			FAT-% of control		SD		t-TEST	
	miR	Stock #	M	F	M	F	M	F
1	mir-986	58959	35	197	na	na	na	na
2	mir-971	58952	44	134	2.7	na	0.020	na
3	mir-263b	58903	47	120	17.6	30.7	0.004	0.060
4	mir-193	58898	50	93	5.1	5.9	0.010	0.245
5	mir-989	58962	53	160	na	11.8	na	0.930
6	mir-1011	58887	53	154	21.6	35.1	0.021	0.669
7	mir-317	58926	55	124	3.4	7.1	0.002	0.965
8	mir-278	58909	58	110	2.6	12.8	0.003	0.500
9	mir-956	58941	58	191	na	29.9	na	0.154
10	mir-995	58965	58	165	2.7	17.9	0.042	0.628
11	mir-967	58948	59	167	20.4	47.9	0.016	0.906
12	mir-9b	58919	60	264	17.6	na	0.100	na
13	mir-1000	58882	60	155	16.3	2.3	0.028	0.619
14	mir-1006	58884	62	147	na	na	na	na
15	mir-932	58939	64	147	7.2	na	0.005	na
16	mir-210	58899	65	105	3.8	7.5	0.025	0.322
17	mir-274	58904	65	105	6.4	24.4	0.028	0.422
18	mir-283	58912	66	170	33.5	52.1	0.069	0.013
19	mir-314	58924	69	122	3.7	14.4	0.010	0.929
20	mir-987	58960	69	185	na	19.5	na	0.009
21	mir-285	58914	70	158	11.9	18.1	0.069	0.732
22	mir-308	58921	70	97	17.4	41.4	0.050	0.021
23	mir-252	58901	74	162	na	12.8	na	0.925
24	mir-307a	58920	78	156	11.4	16.8	0.101	0.661
25	mir-1014	58888	78	133	12.5	10.0	0.140	0.019
26	mir-3	58922	78	249	6.3	30.0	0.243	0.020
27	mir-31b	58929	79	188	2.9	57.5	0.082	0.862
28	mir-994	58964	79	149	7.5	22.0	0.067	0.497
29	mir-929	58936	80	151	7.9	23.8	0.029	0.042
30	mir-276a	58906	80	120	28.5	8.9	0.038	0.840
31	mir-276b	58907	84	151	19.6	9.3	0.312	0.566
32	mir-970	58951	84	190	17.7	42.9	0.325	0.867
33	mir-1017	58889	87	115	30.2	24.0	0.553	0.037
34	mir-304	58918	88	135	4.0	16.7	0.285	0.144
35	mir-10	58880	90	245	24.6	26.7	0.610	0.019
36	mir-990	58963	92	195	9.0	17.8	0.425	0.043
37	mir-965	58946	93	176	2.5	15.2	0.454	0.370
38	mir-124	58891	95	205	7.6	13.8	0.400	0.045
39	mir-284	58913	95	161	na	na	na	na
40	let-7-C	58881	95	216	9.9	na	0.726	na
41	mir-87	58934	95	211	14.9	0.0	0.618	0.036
42	mir-1007	58885	96	186	13.2	12.6	0.691	0.129
43	mir-34	58908	96	171	1.8	11.4	0.200	0.992

44	mir-2a-1	59032	98	228	18.8	2.5	0.914	0.040
45	mir-981	58956	99	154	20.3	9.5	0.936	0.439
46	mir-9c	58967	99	251	na	na	na	na
47	mir-31a	58928	99	181	10.0	12.5	0.900	0.423
48	mir-963	58945	100	181	16.3	105.7	0.984	0.937
49	mir-983-1	58958	100	213	na	na	na	na
50	mir-133	58892	103	215	3.0	3.0	0.435	0.028
51	mir-219	58900	104	149	2.6	2.2	0.716	0.499
52	mir-14	33067	104	197	na	22.7	na	0.207
53	mir-318	58927	106	265	5.0	8.6	0.246	0.002
54	mir-375	58931	106	256	15.1	9.3	0.538	0.004
55	mir-966	58947	108	194	8.8	27.1	0.593	0.645
56	mir-955	58940	109	161	19.1	16.1	0.560	0.067
57	mir-303	58957	112	196	19.2	35.2	0.309	0.119
58	mir-968	58949	114	208	6.7	na	0.239	na
59	mir-1010	58886	115	169	7.8	11.6	0.215	0.282
60	mir-2b-1	58915	124	335	16.9	12.5	0.178	0.000
61	mir-969	58950	149	256	15.0	13.5	0.019	0.001
